# Complete Genome Sequence of *Pelosinus fermentans* JBW45, a Member of a Remarkably Competitive Group of *Negativicutes* in the *Firmicutes* Phylum

**DOI:** 10.1128/genomeA.01090-15

**Published:** 2015-09-24

**Authors:** Kara B. De León, Sagar M. Utturkar, Laura B. Camilleri, Dwayne A. Elias, Adam P. Arkin, Matthew W. Fields, Steven D. Brown, Judy D. Wall

**Affiliations:** a Division of Biochemistry, University of Missouri, Columbia, Missouri, USA; b Graduate School of Genome Science and Technology, University of Tennessee, Knoxville, Tennessee, USA; c Department of Microbiology and Immunology, Montana State University, Bozeman, Montana, USA; d Center for Biofilm Engineering, Montana State University, Bozeman, Montana, USA; e Biosciences Division, Oak Ridge National Laboratory, Oak Ridge, Tennessee, USA; f Physical Biosciences Division, Lawrence Berkeley National Laboratory, Berkeley, California, USA; g Department of Bioengineering, University of California, Berkeley, California, USA

## Abstract

The genome of *Pelosinus fermentans* JBW45, isolated from a chromium-contaminated site in Hanford, Washington, USA, has been completed with PacBio sequencing. Nine copies of the rRNA gene operon and multiple transposase genes with identical sequences resulted in breaks in the original draft genome and may suggest genomic instability of JBW45.

## GENOME ANNOUNCEMENT

*Pelosinus fermentans* JBW45 was isolated from groundwater stimulated for hexavalent-chromium reduction by injection of a polylactate compound (1). *Pelosinus* spp. are found in sites contaminated by heavy metals, explosives, and chlorinated solvents, at low or below-detection levels, but become dominant following nutrient addition *in vitro* (2–14) or *in situ* (1, 15, 16). A strict anaerobe, JBW45 likely resides in sediment-associated, anaerobic microsites (6). Although *Pelosinus* strains have been seen to form spores (17, 18), spore formation has not been documented for JBW45.

Previous genome sequencing of JBW45 with Illumina technology resulted in 98 contigs (1). Draft genome sequences for three other *Pelosinus* spp. from Hanford (A11, B4, and HCF1) and the type strain R7 from Russian kaolin clays are similar to each other but show little synteny with JBW45 or the completed genome of *Pelosinus* sp. UFO1, isolated from Oak Ridge, Tennessee, USA (19–21).

The complete genome sequence of JBW45 was determined with a Pacific Biosciences (PacBio, Menlo Park, California, USA) RSII instrument with P4-C2 chemistry as described previously (22). Two single-molecule real-time (SMRT) cells yielded 1,345,758,432 bases in 202,124 reads with a mean and maximum read length of 6,656 and 35,018 bases, respectively. SMRT analysis version 2.2 and HGAP version 3.0 (PacBio) were used for sequence assembly, which was improved with Pilon (23) and annotated as described previously (1). A single contig with 32-kb overlapping ends was generated, differing only by the presence of a putative transposase gene (JBW_01610). PCR across this region showed the transposase present in 4 of 14 JBW45 colonies, suggesting that the transposase may be actively moving and possibly contributing to evolutionary plasticity (24). Six identical copies (49.6% G+C content) were found in the completed JBW45 genome. This transposase was not found in the completed UFO1 genome. A similar gene (82 to 83% identity) was found as the only gene on a small contig in the draft genomes of A11, B4, HCF1, and R7. JBW45 contains 18 other genes annotated as transposases, many of which occur multiple times in the genome. Transposase and rRNA operon sequences resulted in breaks in assembly of the original JBW45 genome, underscoring the value of longer sequencing technologies, which is consistent with other reports (22, 25, 26).

The final assembly was circularized, resulting in a 5,380,816-bp chromosome with a G+C content of 39.5% and 250-fold sequence coverage. A total of 4,743 protein-coding, 77 tRNA, and 28 rRNA (ten 5S, nine 23S, and nine 16S) genes were identified. Four of the 16S rRNA genes contained a 100-bp insertion, which is consistent with previous findings of intragenomic 16S rRNA gene heterogeneity in UFO1 (21, 27). The average number of bacterial rRNA operons is four (28); however, UFO1 contains fifteen 16S rRNA genes (21). A higher number of rRNA operons may allow rapid adaptation and recovery from the stationary phase (29, 30). This may provide *Pelosinus* spp. with a competitive advantage upon nutrient stimulation.

### Nucleotide sequence accession numbers.

This whole-genome shotgun project has been deposited in DDBJ/ENA/GenBank under the accession number CP010978. The version described in this paper is the first version, CP010978.1.
